# Social network analysis in pigs: impacts of significant dyads on general network and centrality parameters

**DOI:** 10.1017/S1751731119001836

**Published:** 2019-08-15

**Authors:** K. Büttner, I. Czycholl, K. Mees, J. Krieter

**Affiliations:** Institute of Animal Breeding and Husbandry, Christian-Albrechts-University, Olshausenstr. 40, D-24098 Kiel, Germany

**Keywords:** swine, group structure, dominance, mixing, agonistic interactions

## Abstract

In general, one animal is considered dominant over another animal if it has won more fights than its opponent. Whether this difference in won and lost fights is significant is neglected in most studies. Thus, the present study evaluates the impact of two different calculation methods for dyadic interactions with a significant asymmetric outcome on the results of social network analysis regarding agonistic interactions of pigs in three different mixing events (weaned piglets, fattening pigs and gilts). Directly after mixing, all animals were video recorded for 17 (fattening pigs, gilts) and 28 h (weaned piglets), documenting agonistic interactions. Two calculation methods for significant dyads, that is, dyadic interactions with a clear dominant subordinate relationship in which one animal has won significantly more fights than its encounter, were proposed: pen individual limits were calculated by a sign test considering the differences of won and lost fights of all dyadic interactions in each pen; dyad individual limits were determined by a one-sided sign test for each individual dyad. For all data sets (ALL, including all dyadic interactions; PEN or DYAD, including only significant dyads according to pen or dyad individual limits), networks were built based on the information of initiator and receiver with the pigs as nodes and the edges between them illustrating attacks. General network parameters describing the whole network structure and centrality parameters describing the position of each animal in the network were calculated. Both pen and dyad individual limits revealed only a small percentage of significant dyads for weaned piglets (12.4% or 8.8%), fattening pigs (4.2% or 0.6%) and gilts (3.6% or 0.4%). The comparison between the data sets revealed only high Spearman’s rank correlation coefficients (*r_S_*) for the density, that is, percentage of possible edges that were actually present in the network, whereas the centrality parameters showed only moderate *r_S_* values (0.37 to 0.75). Thus, the rank order of the animals changed due to the exclusion of insignificant dyads, which shows that the results obtained from social network analysis are clearly influenced if insignificant dyads are excluded from the analyses. Due to the fact that the pen individual limits consider the overall level of agonistic interactions within each pen, this calculation method should be preferred over the dyad individual limits. Otherwise, too many animals in the group became isolated nodes with zero centrality for which no statement about their position within the network can be made.

## Implications

The determination of significant dyads in agonistic interactions is important in order to obtain the real dominance hierarchy within a group of animals. Only if the amount and the impact of significant dyads on further parameters are known, misinterpretations can be avoided. Thus, the present study presents two different calculation methods for significant dyads and their impact on parameters derived from social network analysis.

## Introduction

In pig husbandry, rehousing of unfamiliar animals is a standard procedure which leads to rank fights, that is, agonistic interactions, between the animals with a variety of expressions of individual behaviour. Therefore, deeper knowledge about the relation between social structure and group stability has potential to improve the management of agonistic interactions related to the practice of rehousing and mixing. Here, social network analysis (Newman, 2010) provides a large number of parameters describing the whole network structure and the position of each individual within the structure which goes beyond the simple dyadic interaction level and offers the ability to precisely capture and quantify social behaviours including indirect connections between the animals (Foister *et al.*, 2018).

Social networks consist of nodes and edges. Nodes represent the animals of each pen and edges are the connections between them (e.g. agonistic interactions, grooming and food competition). Depending on the social interaction which should be investigated, the edges can be undirected (i.e. the direction of the interaction from initiator to receiver is not considered) or directed (i.e. each edge has a clear initiator and a clear receiver). Information about the whole network structure is provided by general network parameters (density, strongly connected components (**SCCs**) and weakly connected components (**WCCs**), and fragmentation) enabling the comparison of different animal groups with each other. Also individual animals can be characterised with the help of centrality parameters (e.g. degree, betweenness closeness) describing the position of each individual within the network.

Hitherto, few studies have applied social network analysis to farm animals. The only reports on livestock have investigated agonistic interactions in pigs (Büttner *et al.*, 2015a and 2015b; Foister *et al.*, 2018), dynamic group structures in dairy cows (Boyland *et al.*, 2016) and behavioural disorders in pigs (Li *et al.*, 2018).

Beside social network analysis, group structure, specifically social hierarchies, can be determined with the help of dominance indices which are calculated from the number of won and lost fights of each dyadic interaction. Here, dominance is defined as a pattern found in repeated agonistic interactions between two animals characterised by the consistent outcome of the agonistic interactions to the advantage of one animal (Drews, 1993). The assumption of the consistent outcome is neglected in the calculations of the usual dominance indices. Here, one animal is dominant over another animal if it has won only one fight more than its opponent (Appleby, 1983; Vries, 1995), which contradicts the definition of dominance according to Drews (1993). Thus, several studies on dominance have claimed that the consistency of the outcome of dyadic interactions has to be tested for significance before further sociometric measures are calculated in order to avoid misinterpretations (Boyd and Silk, 1983; Langbein and Puppe, 2004).

Considering farm animals, only a few studies have included information about significant dyads. On the one hand, empirical measurements have been made (Hunter *et al.*, 1988; Côté, 2000), and on the other hand, objective statistical methods have been used (Martin *et al.*, 1997; Langbein and Puppe, 2004; Puppe *et al.*, 2008) to test the dyadic interactions according to a significant asymmetric outcome. Until now there is a clear lack of knowledge about how the exclusion of insignificant dyads may impact the results of further analyses. To the authors’ knowledge, up to now there are no studies investigating the impact of significant dyads on the results of social network analysis.

Thus, in the present study two different calculation methods for significant dyads are proposed based on pen and dyad individual limits for a significant asymmetric outcome and their impact on parameters derived from social network analysis was investigated. Quantifying the important aspects of group structure with different methodological approaches helps to identify and understand the formation of behavioural patterns and the establishment of stable group structures.

## Material and methods

### Animals and housing

From December 2010 until August 2012, video observation data of pigs in three different rehousing and mixing events (weaned piglets, fattening pigs and gilts) were recorded on the research farm ‘Hohenschulen’ of the Institute of Animal Breeding and Husbandry of the University of Kiel (Germany). The herd consisted of purebred and crossbred animals of the German Landrace and Large White breeds. Information about the different accommodation used for the three observed age groups is given in Table 1. After weaning, the animals were rehoused and mixed in the flat deck pens with an average number of 8.9 ± 0.6 per pen. No animal was acquainted with each other from the farrowing unit. After the flat deck period, the fattening pigs were rehoused and mixed in the fattening pens with an average number of 20.9 ± 1.7 animals per pen. Here, a maximum number of two animals were acquainted with each other from the flat deck pens. In the 22nd week of age, the gilts were moved to the breeding stable with an average number of 20.8 ± 3.4 animals per pen. A maximum of five gilts per pen were already acquainted from the fattening pens.

**Table 1:** Description of housing conditions for the three age groups (weaned piglets, fattening pigs and gilts)

Age group	Housing conditions
Weaned piglets	Flat deck (2.05 m × 1.36 m) Concrete and metal base floor Solid pelleted feed Nipple drinkers
Fattening pigs	Fattening stable (3.25 m × 2.40 m) Half-slatted and half-solid floor Automatic mash feeding machine Nipple drinkers
Gilts	Breeding stable (7.20 m × 5.40 m) Half-slatted and half-solid floor Automatic mash feeding machine Nipple drinkers

### Video observation and agonistic interactions

To achieve a complete overview of each pen, video cameras were mounted at the ceiling which recorded the behaviour of the animals. Due to the fact that previous studies documented a decline in fighting behaviour at night (Stukenborg *et al*., 2011), video observation was paused from 1800 h to 0700 h. Directly after rehousing (at 1200 h), 2.5 days for weaned piglets (28 h of video observation: day 1: 1200 h to 1800 h; day 2: 0700 h to 1800 h; day 3: 0700 h to 1800 h) and 1.5 days for fattening pigs and gilts (17 h of video observation: day 1: 1200 h to 1800 h; day 2: 0700 h to 1800 h) were analysed using the HeiTelPlayer software (Xtralis Headquarter D-A-CH, HeiTel Digital Video GmbH, Kiel, Germany). Three trained observers investigated the agonistic interactions of weaned piglets. The investigation of the agonistic interactions of fattening pigs and gilts were carried out by only one observer who also analysed the videos of weaned piglets. Training to practice the correct definition and detection of agonistic interactions was performed before the beginning of video analysis using unknown video sequences. After the training, the inter-observer reliability was above 90%. To identify animals involved in agonistic interactions, each animal was marked individually on its back. An agonistic interaction was defined as a fight or displacement with physical contact, which was initiated by one pig including aggressive behavioural elements, followed by submissive behaviour performed by the opponent (Langbein and Puppe, 2004). The following aggressive behavioural patterns were recorded: parallel/inverse parallel pressing, head-to-body-knock, head-to-head-knock, biting and physical displacement. A fight started when an animal showed one of the described behavioural patterns which lasted for more than 1s. The end of an agonistic interaction was determined when the pigs were separated for at least 5s after the fight (Samarakone and Gonyou, 2009). For each agonistic interaction, initiator/receiver, winner/loser and duration were recorded. For further analyses, only agonistic interactions with a clear initiator/receiver and winner/loser were used.

### Calculation of significant dyads and resulting data sets

Considering the definition of dominance according to Drews (1993) stating that a dominant relationship between two animals is defined by the consistent outcome of the agonistic interactions to the advantage of one animal, it is claimed that the outcome of each dyadic interaction has to be tested for significance in order to avoid misinterpretations of further analyses (Boyd and Silk, 1983). Thus, in the present study, two different calculation methods for the limits of significant dyads were carried out, considering pen and dyad individual limits.

#### Pen individual limits

For the calculation of the pen individual limits, a one-sided sign test was used, which takes the outcome of all dyadic interactions into account. It has the null hypothesis that the median of the differences in the number of agonistic interactions, that is, animals with higher number of fights won minus animals with lower number of fights won, of the whole group of animals is zero. The 95% confidence interval is used to get the pen individual limits. The one-sided sign test was calculated with the function SIGN.test() from the R package BSDA (Arnholt and Evans, 2017).

#### Dyad individual limits

For the dyad individual limits, each dyad was tested individually for a significant asymmetric outcome independent of the other dyadic interactions in the group by using a one-sided sign test (Dixon and Mood, 1946) following the methodology used in Langbein and Puppe (2004). The significance limits of this test lead to the result that at least five agonistic interactions with a strictly unidirectional outcome are required to reach significance at an *α* level of 0.05, that is 5 wins *v*. 0 defeats. Further significant ratios of wins to defeats are 5 : 0, 6 : 0, 7 : 0, 7 : 1 and so on.

#### Resulting data sets

For the present study, the following data sets were created and analysed. Data set ALL included all agonistic interactions observed. Based on ALL, significant dyads were calculated. The data set PEN comprised significant dyads determined with pen individual limits. The data set DYAD comprised significant dyads determined with dyad individual limits. Basic information about all data sets is given in Table 2.

**Table 2:** Basic information after 17 h (28 h; only for weaned piglets) of video observation for the three data sets (ALL, including all dyadic interactions; PEN or DYAD, including only significant dyadic interactions according to pen or dyad individual limits) for all age groups (weaned piglets, fattening pigs and gilts)

Data set	Number of pens	Number of animals	Mean ± SD number of animals/network	Number of fights	Mean ± SD number of fights/animal
Weaned piglets					
ALL	93 (93)	829 (829)	8.9 ± 0.6 (8.9 ± 0.6)	5088 (7620)	12.3 ± 11.1 (18.4 ± 17.6)
PEN	90 (92)	806 (820)	9.0 ± 0.5 (8.9 ± 0.6)	2151 (3351)	5.5 ± 8.3 (8.2 ± 12.5)
DYAD	53 (61)	474 (548)	8.9 ± 0.5 (9.0 ± 0.5)	1228 (2495)	5.9 ± 9.3 (9.1 ± 14.4)
Fattening pigs					
ALL	26	543	20.9 ± 1.7	1611	5.9 ± 4.6
PEN	26	543	20.9 ± 1.7	552	2.0 ± 3.0
DYAD	3	60	20 ± 2.7	19	0.6 ± 2.0
Gilts					
ALL	12	249	20.8 ± 3.4	665	5.3 ± 4.6
PEN	12	249	20.8 ± 3.4	209	1.7 ± 2.7
DYAD	1	22	22	5	0.5 ± 1.5

### Social network analysis

In this study, ALL, PEN and DYAD were used in order to build social networks containing directed edges pointing from the initiator to the receiver of an agonistic interaction. Through these edges, the nodes are connected with each other over paths representing either direct or indirect connections between the nodes. In directed networks, each path has to follow the direction of edges. If nodes have no ingoing or outgoing edges, they are referred to as isolated nodes. Social network analysis offers a standardised methodology to calculate general network or centrality parameters (Newman, 2010). General network parameters, such as density, SCC and WCC or fragmentation, enable the comparison of different groups of animals and the temporal variation within the same group of animals over different time periods. Due to the focus on each individual animal, centrality parameters help to quantify the extent to which an individual is central in their group and enable the detection of key individuals which play a central role in the social structure of the whole group. A summary of the calculated general network and centrality parameters is illustrated in Table 3. All calculations concerning the social network analysis were performed using the Python module NetworkX (Hagberg *et al.*, 2008).

**Table 3:** Description of the general network and centrality parameters calculated for the social networks of pigs

Parameter	Description
General network parameters	
Density	Amount of agonistic interactions between animals that are present related to the number of agonistic interactions that are possible (Newman, 2010)
WCC	Two animals are part of the same WCC if they are connected by at least one path (direct or indirect) through the network neglecting the direction of the edges (Kao *et al.*, 2006)
SCC	Two animals are part of the same SCC if they are connected by at least one directed path (direct or indirect) through the network; that is, in this case the direction of the edges are taken into account (Kao *et al.*, 2006)
Fragmentation	Number of WCCs in relation to the number of nodes in a network (Borgatti, 2003)
Centrality parameters	
In- and out-degree	Number of ingoing (in-degree) or outgoing (out-degree) agonistic interactions. In the winner loser network, in-degree describes the number of fights lost and the out-degree describes the number of fights won (Newman, 2010)
Betweenness	Measures the extent to which an animal lies on paths between other animals (Freeman, 1977)
Ingoing and outgoing closeness	Mean distance from all other reachable animals to one specific animal (ingoing closeness) or mean distance from one animal to all other reachable animals (outgoing closeness) (Sabidussi, 1966)

WCC=weakly connected component; SCC=strongly connected component.

### Impact of age group on the number of agonistic interactions

A significant difference in the sum of agonistic interactions between the three observed age groups (weaned piglets, fattening pigs and gilts) using 17 h of video observation was estimated with non-parametric Kruskal–Wallis tests using the statistical software SAS 9.4 (SAS® Institute Inc., 2013). To test pairwise differences between the age groups subsequent Dunn’s *post hoc* tests were carried out.

### Comparison of general network and centrality parameters between the data sets

In order to estimate the temporal development of the parameters derived from social network analysis, they were calculated using increasing time window lengths over the whole observation period separated for each age group. Thus, the first time window comprised the observation times day 1: 1200 h until day 1: 1300 h (includes 1 h), the second time window comprised the observation times day 1: 1200 h until day 1: 1400 h (includes 2 h) and so on until the whole observation period from day 1: 1200 h until day 3: 1800 h (includes 28 h) for weaned piglets or until day 2: 1800 h (includes 17 h) for fattening pigs and gilts was covered. For each time window length, a Spearman’s rank correlation coefficient (*r_S_*) using the statistical software package SAS 9.4 (SAS® Institute Inc., 2013) was calculated between the general network and centrality parameters for each of the data sets in order to evaluate the effect of the exclusion of dyads with an insignificant asymmetric outcome of the agonistic interactions on the results of social network analysis. To determine significant differences between the data sets, non-parametric Kruskal–Wallis tests using the statistical software package SAS 9.4 (SAS® Institute Inc., 2013) were carried out. To test pairwise differences between the results of the data sets, subsequent Dunn’s *post hoc* tests were performed.

## Results

### Significant dyads calculated according to pen and dyad individual limits

In Table 4, the percentages of significant dyads based on the two calculation methods are illustrated for all age groups. Weaned piglets revealed a clearly higher amount of significant dyads for both calculation methods compared to fattening pigs and gilts. These results are in accordance with the number of fights per animal and hour regarding ALL. Here, weaned piglets fought significantly more often (12.3 fights/animal per h) compared to fattening pigs (5.9 fights/animal per h) and gilts (5.3 fights/animal per h) (*P*<0.05). No significant differences in the number of agonistic interactions could be obtained between fattening pigs and gilts.

**Table 4:** Mean (±SD) percentage (%) of significant dyads calculated based on pen and dyad individual limits for a significant asymmetric outcome after 17 h (28 h; only for weaned piglets) of video observation for all age groups (weaned piglets, fattening pigs and gilts)

	Percentage of significant dyads	
	Pen individual limits	Dyad individual limits
Weaned piglets	12.4 ± 6.8^a^ (15.2 ± 7.7^a^)	8.8 ± 7.3^b^ (13.3 ± 11.2^b^)
Fattening pigs	4.2 ± 2.0^a^	0.6 ± 0.2^b^
Gilts	3.6 ± 2.9^a^	0.4^b^

^a,b^Significant differences (*P*<0.05) between the number of significant dyads according to pen or dyad individual limits are indicated by different letters.

Due to the low number of significant dyads obtained from the calculation method based on dyad individual limits for fattening pigs and gilts (Table 2), only a comparison between ALL and PEN are further carried out for these age groups.

### Network visualisations

**Figure 1:**
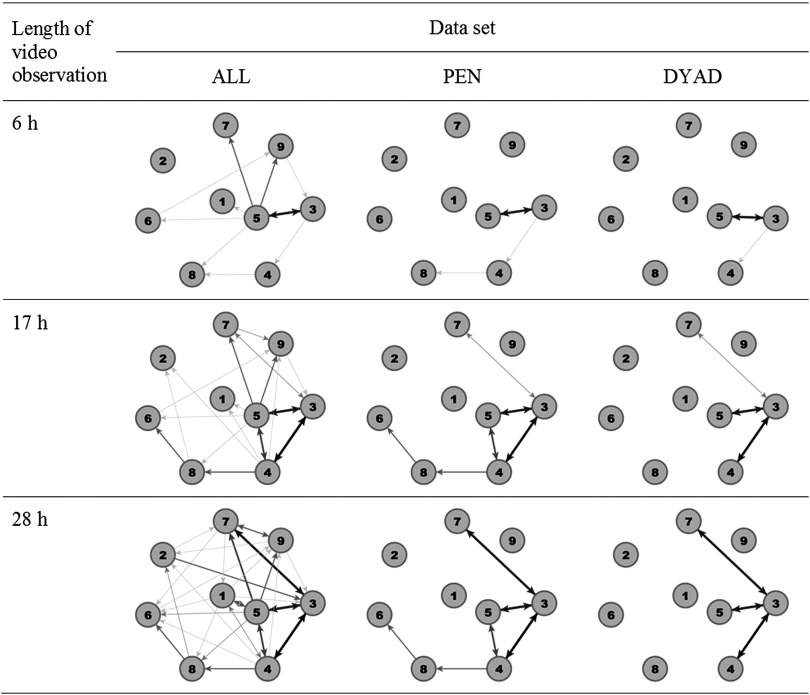
Example network visualisation for one pen of weaned piglets after 6 h (end of video observation at day 1), 17 h (end of video observation at day 2) and 28 h (end of video observations at day 3) for all data sets (ALL, including all dyadic interactions; PEN or DYAD, including only significant dyadic interactions according to pen or dyad individual limits). Thicker and darker edges illustrate more agonistic interactions.

As an example, Figure 1 illustrates the network of one pen of weaned piglets after 6 h (end of video observation at day 1), 17 h (end of video observation at day 2) and 28 h (end of video observations at day 3) based upon the three data sets. The network built from ALL revealed the highest number of edges for each time window followed by PEN and DYAD. After 28 h, in ALL, all nodes were connected with each other, that is, there were no isolated nodes, whereas in PEN three animals and in DYAD five animals were isolated.

### Descriptive statistics of the general network and centrality parameters

#### General network parameters

**Figure 2:**
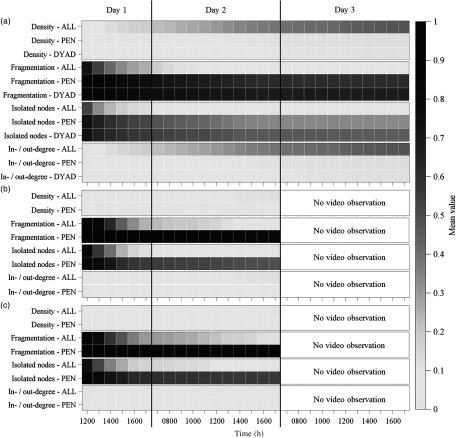
Development of mean density, fragmentation, amount of isolated nodes and in-/out-degree with increasing time window length for all age groups (weaned piglets (a), fattening pigs (b) and gilts (c)) and all data sets (ALL, including all dyadic interactions; PEN or DYAD, including only significant dyadic interactions according to pen or dyad individual limits). Standardised in- /out-degree centrality (range: 0 to 1) are illustrated.

Figure 2 illustrates the temporal development of then mean density, fragmentation and amount of isolated nodes considering the networks of the three age groups and data sets.

For weaned piglets considering ALL, the mean density increased with increasing time interval until it reached a mean value of 0.48 ± 0.17 after 28 h. PEN and DYAD revealed also an increase in density, but at a much lower level. They reached after 28 h only values of 0.13 ± 0.07 and 0.12 ± 0.10. Although only about 50% of the edges were present in the networks of ALL, fragmentation reached values below 0.30 after 5 h and decreased further until it reached a value close to 0 after 28 h. This could also be confirmed by the results of the largest WCC size. After 28 h, the largest WCC contained 99.7 ± 1.9% of the animals in each pen. Also the largest SCC size revealed an increase with increasing time window size. After 28 h, 90.3 ± 13.5% of the animals were part of the largest SCC. ALL contained at the end of the observation period only 0.4 ± 1.2% isolated nodes. PEN and DYAD showed also a decrease in fragmentation but with higher mean values of 0.61 ± 0.29 and 0.68 ± 0.33 after 28 h, indicating that the network consists of more disconnected network components. This again is also reflected by largest WCC size with 59.2 ± 22.9% (PEN) and 50.8 ± 27.6% (DYAD) of the animals in each pen. The largest SCC size showed lower values with 44.3 ± 23.7% (PEN) and 43.3 ± 25.9% (DYAD). PEN contained 34.0 ± 21.2% of isolated nodes after 28 h. The amount of isolated nodes was even higher in DYAD with 45.6 ± 27.3%.

The results for the density of fattening pigs and gilts revealed similar values compared to weaned piglets, but at a much lower level. The mean density for ALL reached only values of 0.12 ± 0.02 for fattening pigs and 0.11 ± 0.04 for gilts after 17 h of video observation. For PEN, the values were even lower with 0.03 ± 0.01 (fattening pigs) and 0.02 ± 0.02 (gilts). Also fragmentation and largest WCC or SCC sizes for fattening pigs and gilts showed similar values compared to weaned piglets. The mean fragmentation for ALL reached also values close to 0 after 17 h. Additionally, the clear drop below a fragmentation of 0.30 after 5 h could be observed in both age groups. This could also be confirmed by the results of the largest WCC and SCC sizes. After 17 h, the largest WCC contained 97.7 ± 3.8% or 93.9 ± 7.5% of the animals for fattening pigs or gilts. Also the largest SCC size showed an increase with increasing time window size. After 17 h, 61.1 ± 16.7% or 54.7 ± 22.3% of the animals were part of the largest SCC for fattening pigs or gilts. For ALL, fattening pigs contained on average 1.7 ± 2.8% isolated nodes at the end of the observation period. For gilts, the amount of isolated nodes was slightly higher at 5.1 ± 5.1%. At the end of the observation period, PEN also revealed a relatively high fragmentation of 0.85 ± 0.14 (fattening pigs) or 0.87 ± 0.20 (gilts). This was also confirmed by the results of the largest WCC size. Only 34.8 ± 18.2% or 29.4 ± 22.1% of the animals belonged to the largest WCC for fattening pigs and gilts. The largest SCC size was even lower at 17.5 ± 8.8% for fattening pigs and 13.2 ± 5.5% for gilts. Here, 49.2 ± 13.7% (fattening pigs) and 57.2 ± 18.5% (gilts) of isolated nodes were obtained after 17 h.

#### Centrality parameters

Figure 2 illustrates the temporal development of the mean in- or out-degree for the networks built from the three data sets. Table 5 illustrates the descriptive statistics for all centrality parameters. Independent of the age group, ALL showed always the highest mean centralities. However, especially for weaned piglets the maximum centralities changed only slightly between the data sets, illustrating that the animals with the most agonistic interactions remained in the network. Furthermore, due to the exclusion of insignificant dyads, animals with only few agonistic interactions became isolates with zero centrality lowering the mean values for PEN and DYAD.

**Table 5:** Descriptive statistics of the centrality parameters for all age groups (weaned piglets, fattening pigs and gilts) and data sets (ALL, including all dyadic interactions; PEN or DYAD, including only significant dyadic interactions according to pen or dyad individual limits)

	Data sets					
	ALL		PEN		DYAD	
	Mean ± SD	Max	Mean ± SD	Max	Mean ± SD	Max
Weaned piglets (28 h of video observation)						
In-degree	3.8 ± 1.9^a^	9	1.0 ± 1.1^b^	6	0.9 ± 1.2^b^	7
Out-degree	3.8 ± 2.3^a^	9	1.0 ± 1.3^b^	7	0.9 ± 1.3^b^	8
Betweenness	0.08 ± 0.10^a^	0.64	0.03 ± 0.09^b^	0.66	0.03 ± 0.08^b^	0.58
Ingoing closeness	0.60 ± 0.19^a^	1.00	0.18 ± 0.18^b^	0.80	0.16 ± 0.20^b^	0.89
Outgoing closeness	0.61 ± 0.24^a^	1.00	0.18 ± 0.20^b^	0.89	0.16 ± 0.21^b^	1.00
Weaned piglets (17 h of video observation)						
In-degree	3.1 ± 1.7^a^	8	0.8 ± 1.0^b^	6	0.6 ± 0.8^c^	4
Out-degree	3.1 ± 2.3^a^	9	0.8 ± 1.1^b^	7	0.6 ± 1.0^c^	8
Betweenness	0.08 ± 0.10^a^	0.59	0.03 ± 0.08^b^	0.64	0.01 ± 0.05^c^	0.52
Ingoing closeness	0.50 ± 0.20^a^	1.00	0.14 ± 0.16^b^	0.75	0.09 ± 0.12^c^	0.52
Outgoing closeness	0.51 ± 0.27^a^	1.00	0.14 ± 0.18^b^	0.88	0.09 ± 0.15^c^	1.00
Fattening pigs (17 h of video observation)						
In-degree	2.4 ± 1.6^a^	9	0.6 ± 0.8^b^	4	–	–
Out-degree	2.4 ± 2.3^a^	11	0.6 ± 0.9^b^	7	–	–
Betweenness	0.05 ± 0.07^a^	0.37	0 ± 0.02^b^	0.21	–	–
Ingoing closeness	0.25 ± 0.11^a^	0.51	0.04 ± 0.06^b^	0.29	–	–
Outgoing closeness	0.25 ± 0.18^a^	0.70	0.04 ± 0.07^b^	0.47	–	–
Gilts (17 h of video observation)						
In-degree	2.1 ± 1.6^a^	9	0.5 ± 0.8^b^	4	–	–
Out-degree	2.1 ± 2.1^a^	15	0.5 ± 0.9^b^	8	–	–
Betweenness	0.05 ± 0.07^a^	0.33	0 ± 0.01^b^	0.10	–	–
Ingoing closeness	0.22 ± 0.12^a^	0.55	0.03 ± 0.06^b^	0.33	–	–
Outgoing closeness	0.23 ± 0.17^a^	0.94	0.03 ± 0.06^b^	0.51	–	–

^a,b,c^Significant differences (*P*<0.05) between the mean results of the different data sets are indicated by different letters.

### Comparison of the general network and centrality parameters between the data sets

#### General network parameters

For weaned piglets, the *r_S_* values of the density between the data sets showed consistent highly positive values (0.73 to 0.92, *P*<0.05) over all considered time window lengths, that is, for 1 h until 28 h after regrouping. Fragmentation and largest WCC or SCC sizes revealed only high *r_S_* values for all time window lengths after regrouping between PEN and DYAD (fragmentation: 0.85 to 0.91; largest WCC size: 0.76 to 0.87; largest SCC size: 0.83 to 0.94). Both correlations, ALL *v*. PEN and ALL *v*. DYAD, showed a clear drop with increasing time window length. The significant *r_S_* values for the fragmentation decreased from 0.80 to 0.21 or 0.74 to 0.30. After 17 h for the comparison between ALL and PEN and after 11 h for the comparison between ALL and DYAD, the significance was no longer apparent. Similar values were obtained for the *r_S_* values of the largest WCC size. The *r_S_* values considering all time window lengths were slightly higher for the largest SCC size. Here, the *r_S_* values dropped from 0.83 to 0.27 (ALL *v.* PEN) and 0.77 to 0.40 (ALL *v.* DYAD) with increasing time window length.

For fattening pigs and gilts, the *r_S_* values of the density between ALL and PEN were comparable to those for weaned piglets and ranged between 0.64 and 0.94. Also a clear drop of the *r_S_* values with increasing time window length for fragmentation, largest WCC and SCC size between ALL and PEN were obtained. Here, the significance of the *r_S_* values was only apparent for the first 5 h of video observation for fragmentation and largest WCC size. The significant *r_S_* values for fragmentation decreased from 0.90 to 0.42 (fattening pigs) or 0.96 to 0.73 (gilts) with increasing time window length. The *r_S_* values of largest SCC size showed similar results. The *r_S_* values for the largest SCC size dropped from 1.00 to 0.42 (fattening pigs) or 0.94 to 0.48 (gilts) with increasing time window length.

#### Centrality parameters

For weaned piglets, the *r_S_* values of the centrality parameters between ALL and PEN or DYAD revealed consistent positive values (0.48 to 0.75, *P*<0.05). The only exception was the betweenness which showed a decline of *r_S_* values with increasing time window length (0.12 to 0.54). The highest *r_S_* values were obtained for the centrality parameters between PEN and DYAD (0.66 to 0.87). In each data set comparison, centrality parameters focussing on outgoing agonistic interactions (out-degree, outgoing closeness), that is, attacks delivered, revealed slightly higher *r_S_* values compared to centrality parameters based on ingoing agonistic interactions (in-degree, ingoing closeness), that is, being attacked, and betweenness.

For fattening pigs and gilts, the correlation coefficients of the centrality parameters between ALL and PEN had positive *r_S_* values (0.37 to 0.68), similar to weaned piglets. The highest *r_S_* values were obtained for out-degree and outgoing closeness followed by in-degree and ingoing closeness. Betweenness revealed the lowest *r_S_* values.

## Discussion

In the present study, three data sets were generated containing all dyadic interactions (ALL), significant dyadic interactions according to the pen individual limits (PEN) and significant dyadic interactions according to dyad individual limits (DYAD) in order to evaluate the impact of insignificant dyads on parameters derived from network analysis.

### Significant dyads calculated according to pen and dyad individual limits

Only for a small amount of dyadic interactions, a significant asymmetric outcome was found. These results were in accordance with the number of agonistic interactions observed in the three age groups; that is, weaned piglets had significantly more fights compared to fattening pigs and gilts. Adult animals can better evaluate their own fighting ability and that of their pen mates (Puppe *et al.*, 2008) and developed additional behavioural mechanisms to avoid overt agonistic interactions (e.g. avoidance order (Jensen, 1982)). Furthermore, experiences from previous fights reinforce future behaviour, which is in accordance with the study of D’Eath (2004), who described that the animals gained confidence in the rank position they already achieved. There are only few studies which provide information about the percentage of significant dyads in pigs. However, Puppe *et al.* (2008), who analysed the agonistic interactions of pigs in different age groups, stated that the amount of significant dyads was about 38% for weaned piglets, 54% for fattening pigs and 23% for sows which is clearly higher compared to the present results. Also the study of Langbein and Puppe (2004) investigated the agonistic interactions in one group of pigs directly after weaning and after rehousing in the fattening stable, and revealed a higher amount of significant dyads at 35% for weaned piglets. Only the amount of significant dyads for fattening pigs of about 3% in the study of Langbein and Puppe (2004) was comparable to the results for significant dyads obtained with pen individual limits. In both studies, significant dyads were determined with the help of a two-sided sign test for each dyadic interaction which resembles the dyad individual limits presented in this study. Although the calculation methods differed slightly, this cannot be the only explanation. The results of the present study demonstrated considerable variation in the amount of significant dyads between the individual pens which points to a large variation in the behavioural patterns of each single group of animals. This can also be seen as explanation between the present results and the results obtained from literature. Also other behavioural studies focussing on behavioural disorders, that is, tail biting, showed large differences between individual pens (Zonderland *et al.*, 2008; Veit *et al.*, 2017). Although the animals were kept in the same husbandry with identical feed and enrichment material, some of the pens showed tail biting and others not. Here, also individual differences of each animal should be considered to explain these differences. Thus, the inclusion of other impact factors in the analyses such as group composition, body weight, recent social experiences, health status and management procedures (Martin *et al.*, 1997; Andersen *et al.*, 2004) is recommended to achieve deeper insights into the formation of specific behavioural patterns and to find explanations for the variation in behaviour between individual pens.

The differences in the amount of significant dyads between weaned piglets and fattening pigs or gilts can be explained by the larger group sizes in the older age groups. In larger groups, there are fewer fights after mixing (Marchant-Forde and Marchant-Forde, 2005) and also a lower probability that all animals are involved in rank fights with any other animal in the pen, that is, the probability for a higher amount of unknown dyads increases in larger groups (Klass and Cords, 2011). Due to the fact that with increasing group size generally also the pen size increases in conventional pig husbandry, pen size has also to be considered for the differences in the amount of significant dyads between weaned piglets and both older age groups. Larger pen sizes provide more means of escape, which allows subordinate animals to avoid overt agonistic interactions with higher ranked animals (Hemsworth *et al.*, 2013; Rault, 2017). In the present study, the familiarity between the animals increased with each mixing event which could also be an explanation for the lower amount of significant dyads in the older age groups. The higher the level of familiarity within a group of animals, the fewer agonistic interactions can be observed (Arey and Franklin, 1995). Here, the rank order between the animals is already settled, which makes further overt agonistic interactions no longer necessary.

### Social network analysis

Due to the very low percentage of significant dyads for fattening pigs and gilts, a comparison of the general network and centrality parameters between all three data sets was only possible for the weaned piglets. Although the amount of significant dyads varied between the two calculation methods, the results of the social network analysis remained relatively stable between the two methods (high *r_S_* values for the comparison between PEN and DYAD). However, considering the comparison between ALL and PEN or DYAD only high *r_S_* values were present for density, whereas fragmentation and largest WCC and SCC sizes revealed a clear decrease in the *r_S_* values with increasing time window size. This indicates that the network structure, especially the formation of network components (WCC and SCC), is influenced by the inclusion of insignificant dyads. The inclusion of insignificant dyads also increased the probability of forming larger connected components. Furthermore, the centrality parameters showed only moderate *r_S_* values indicating that the rank order of the animals based on these parameters was influenced by the exclusion of insignificant dyads, whereby higher *r_S_* values were obtained for out-degree and outgoing closeness, that is, centrality parameters reflecting an active behaviour, compared to in-degree and ingoing closeness, that is, centrality parameters reflecting a passive behaviour (Büttner *et al.*, 2015a and 2015b). It has to be borne in mind that all centrality parameters were measured for the network based on information of the initiator and the receiver of the fight without information about the further sequence of behavioural patterns. This relation is of special importance when the social structure is analysed. Here, the natural behaviour of the animals is influenced by environmental impacts made by humans, for example, limited space allowance or predetermined pen mates (Koene and Ipema, 2014). In the wild, a subordinate animal can more easily avoid an agonistic interaction compared to the artificial environment of a stable with few means of escape (Büttner *et al.*, 2015b). Also other studies stated that with increased available space the amount of agonistic interactions is reduced (Remience *et al.*, 2008; Hemsworth *et al.*, 2013; Rault, 2017). Thus, it can be assumed that the restraints made by humans impact the agonistic interactions in pigs which lead to an increase in the total amount of agonistic interactions. If two groups of animals, one with enough means of escape and the other with limited available space are compared, the total number of agonistic interactions should be increased in the group with the limited space which does not automatically imply that also the amount of significant dyads is increased. Also Remience *et al.* (2008) stated that the number of nonreciprocal agonistic interactions, that is, the attacked animal retaliated, is increased with limited available space. Thus, these nonreciprocal agonistic interactions increase the total amount of agonistic interactions but may not attribute to significant dyads which will lower the amount of significant dyads in the group. This was not analysed in the present study but should be included in further investigations in order to obtain further characteristics of significant dyads regarding the sequence of behavioural patterns performed.

### Determination of significant dyads

There was a considerable variation in the number of agonistic interactions between the different pens. Due to this large variation, it is questionable if averaging over different groups of animals by calculating the amount of significant dyads with dyad individual limits is the appropriate method. Here, independent of the total amount of agonistic interactions within a group, rigid boundaries for the limits of significant dyadic interactions were assigned, which may lead to an underestimation of their number. Thus, the pen individual limits provide a better refinement due to the fact that the overall level of agonistic interactions within each group is taken into account.

Moreover, it has to be borne in mind that the number of isolated nodes increased for PEN and even more for DYAD. Thus, there are an increasing number of animals with zero centrality values for which no position within the network could be determined and which potentially lowered the calculated *r_S_* values. Furthermore, for animals which provide a zero centrality, that is, isolated nodes, no conclusions about their position within the network can be drawn which can be problematic if the whole group of animals should be characterised.

In the literature, few studies on farm animals have included information about significant dyads. Langbein and Puppe (2004) and Puppe *et al.* (2008) used a two-sided sign test in order to test for a significant asymmetric outcome of each individual dyad. In Martin *et al.* (1997), the dominance relationships of hens were analysed. Here, a hen was said to be dominant when an agonistic asymmetry between two animals could be recorded for six consecutive agonistic interactions. Hunter *et al.* (1988) defined a sow as dominant over another sow if at least two agonistic interactions with the same outcome were present and whenever reversals were present, a ratio of 4 : 1 was necessary to assume dominance of one sow.

For wild species, also the amount of significant dyads of agonistic interactions is determined using a two-sided binomial test similar to the study of Langbein and Puppe (2004). In Barkan *et al.* (1986), the social dominance in communal Mexican jays (*Aphelocoma ultramarina*) are determined. Here, for two observation years, 61% and 78% of the dyadic interactions showed a significant asymmetric outcome. In contrast to this, in the study of Tarvin and Woolfenden (1997), which analysed the patterns of dominance and aggressive behaviour in Blue Jays (*Cyanocitta cristata*) at a feeder, only 10% of significant dyads could be obtained. Slightly higher amounts with 19% of significant dyads were found in the study of Post (1992), who studies the dominance and mating success in male boat-tailed grackles (*Quiscalus major*). These three studies on birds illustrate clearly the huge variation between different study groups similar to the huge variation between each pen and the different age groups found in the present study. All these studies used only the significant dyads for further determination of the dominance hierarchy within the group so that no statement about the impact of the exclusion of insignificant dyads can be made.

In addition to the different approaches to define significant dyadic interactions, it also has to be considered that different behaviours also have a different meaning during the establishment of social relationships between animals. In literature, different levels of contest escalation are defined in order to evaluate the intensity of the aggressive behaviour: from display or threat to pushing without damaging, followed by biting which then escalates to fights illustrating the highest level of escalation (Ewbank and Bryant, 1972; Camerlink *et al.*, 2016). Especially threatening behaviour without physical contact between the contestants is often excluded from the analyses as it was done in the present study. However, this might lead to a loss of important information about the social structure in the group. Usually contest escalations are avoided due to the high costs of energy and the high probability to get injured (Lane and Briffa, 2017). Here, agonistic interactions may end without escalating into a severe fight or even before the opponents have physical contact with each other (i.e. non-damaging threat) (Jensen, 1982; Camerlink *et al.*, 2016). Hence, it might be advantageous to include more subtle behavioural patterns in the ethogram for a more detailed picture of the behaviour. This, however, would again increase the complexity of video observation and the probability of erroneous or missed detection of the specified behaviours.

Due to the fact that there is yet no standardised way for the determination of dyadic interactions with a significant asymmetric outcome, future studies should include a detailed description of the definition of significant dyads. Furthermore, all calculations should be carried out using the data sets containing all dyadic interactions and containing only significant dyads in order to be able to evaluate the impact of significant dyads on the outcome of the analyses.

## Conclusion

In the present study, the impact of two different calculation methods for dyadic interactions with a significant asymmetric outcome on the results of social network analysis was evaluated. Both pen and dyad individual limits resulted in only a small percentage of significant dyads for all age groups. The comparison between the data sets showed high *r_S_* values only for density. For the centrality parameters, only moderate *r_S_* values were present, implying that the rank order of the centrality parameters was influenced by the exclusion of insignificant dyads. This impact became even more prominent for fragmentation and largest WCC and SCC sizes characterising the whole network structure. Furthermore, due to the exclusion of insignificant dyads the amount of isolated animals, that is, animals with zero centrality, increased significantly. Pen individual limits should be preferred over dyad individual limits due to the fact that the overall level of agonistic interactions within each pen is considered.

## Software and data repository resources

Data are available from the authors upon reasonable request.
